# Anti-inflammatory potential of ellagic acid, gallic acid and punicalagin A&B isolated from *Punica granatum*

**DOI:** 10.1186/s12906-017-1555-0

**Published:** 2017-01-14

**Authors:** Lamees A. BenSaad, Kah Hwi Kim, Chin Chew Quah, Wee Ric Kim, Mustafa Shahimi

**Affiliations:** 1 Department of Physiology, Faculty of Medicine, Malaysia Institute of Medical Research, Kuala Lumpur, 50603 Malaysia; 2 University College Lincoln, Petaling Jaya, 47301 Malaysia; 3 Malaysia Institute of Medical Research, Kuala Lumpur, 50603 Malaysia

**Keywords:** Inflammation, *Punica granatum*, Cytotoxicity, Cytokines, Ellagic acid, Gallic acid, Punicalagin

## Abstract

**Background:**

*Punica granatum* (pomegranate), an edible fruit originating in the Middle East, has been used as a traditional medicine for treatment of pain and inflammatory conditions such as peptic ulcer. The numerous risks associated with nonsteroidal anti-inflammatory drugs (NSAIDs) for treatment of pain and inflammation give rise to using medicinal herbs as alternative therapies. This study aimed to evaluate the anti-inflammatory effect of isolated compounds from the ethyl acetate (EtOAc) fraction of *P. granatum* by determination of their inhibitory effects on lipopolysaccharide (LPS), stimulated nitric oxide (NO), prostaglandin E2 (PGE-2), interleukin-6 (IL-6) and cyclooxxgenase-2 (COX-2) release from RAW264.7 cells.

**Methods:**

The compounds ellagic acid, gallic acid and punicalagin A&B were isolated from EtOAc by high performance liquid chromatography (HPLC) and further identified by mass spectrometry (MS). The inhibitory effect of ellagic acid, gallic acid and punicalagin A&B were evaluated on the production of LPS-induced NO by Griess reagent, PGE-2 and IL-6 by immunoassay kit and prostaglandin E2 competitive ELISA kit, and COX-2 by Western blotting.

**Results:**

Ellagic acid, gallic acid and punicalagin A&B potentially inhibited LPS-induced NO, PGE-2 and IL-6 production.

**Conclusion:**

The results indicate that ellagic acid, gallic acid and punicalagin may be the compounds responsible for the anti-inflammatory potential of *P. granatum*.

## Background

Inflammatory diseases such as arthritis, asthma, allergic rhinitis and eczema have increased throughout the world and are treated with conventional anti-inflammatory drugs such as steroidal anti-inflammatory drugs and NSAIDs [1]. However, their prolonged use may produce severe side effects which may sometimes be lethal [2].

Therefore, it is important to develop novel and organic anti-inflammatory agents with minimal adverse effects. Pomegranate fruits are widely consumed worldwide. In Libya, it is traditionally regarded as a life giving fruit and used to treat conditions such as gastric ulcers. Anti-inflammatory potential of different varieties of pomegranate has been reported [3–5]. More interestingly, a recent study reported that the extract of pomegranate produced gastro protection along with its anti-inflammatory effect [6].

Apart from its anti-inflammatory potential, pomegranate also earned its medicinal value with a number of proven potentials like anti-lipoperoxidation [7], anti-ulcer [8, 9], and anti-oxidation [10, 11]. Another phenomenal potential of pomegranate is attenuating neurogenic pain [12]. Pomegranate could successfully attenuate tibial and sural nerve transection (TST) induced behavioral alterations, including cold, mechanical and heat hyperalgesia; dynamic mechanical allodynia and cold allodynia. The same study demonstrated a significant reduction in tumor necrosis factor alpha (TNF-α) [12]. Furthermore, pomegranate is useful in minimizing conditions like cancer [13]. Ellagic acid, gallic acid and punicalagin A&B isolated from the EtOAc fraction of the ethanol whole fruit extract have been investigated for their potential inhibitory effects in vitro using LPS-stimulated macrophages. LPS is a potent activator of macrophages, which produce a variety of pro-inflammatory mediators such as NO, PGE-2 and interleukins [14–16].

LPS is one of the best characterized stimuli to induce up-regulation of pro-inflammatory proteins such as COX-2 and inducible NO. Inducible COX-2 is responsible for the high prostaglandin levels observed in inflammatory pathology. A number of inflammatory stimuli such as LPS and pro-inflammatory cytokines activate immune cells to up-regulate such inflammatory states. Therefore, these stimuli are useful targets in the development of new anti-inflammatory drugs [17, 18].

Although there are previous reports on anti-inflammatory effect of *P.granatum* the exact compounds involved have not been identified yet. Therefore, the objective of the present study was to study the effect of gallic acid, ellagic acid and punicalagin isolated from EtOAc fraction of *P. granatum* on mediators of inflammation invitro.

## Methods

### Plant material

Fresh pomegranate fruits, *P. granatum* from Libya, were collected in the fall of 2010 from an orchard in the region of Tripoli, Libya. The sample was identified by Professor AbdulRazag Sheriff, a taxonomist from the Faculty of Science, University of Tripoli. A voucher specimen number 01563 was deposited at the Herbarium at University of Tripoli.

### Chemicals and reagents

All chemicals used were of analytical grades, with purity (determined by GC) of minimum 99.0%, unless otherwise specified. Ethanol, HPLC-grade methanol and sulphuric acid (98%) were purchased from RCI Labscan (Thailand). Phosphoric acid (HPLC-grade, 85–90%) were purchased from Sigma FLUKA (Germany). Bradford reagent, griess reagent, (modified), L-glutamine, LPS (*E.coli*) and sodium pyruvate were purchased from Sigma (USA). RAW264.7 (ATCC® TIB-71™) cells were purchased from ATCC (USA). Dulbecco’s Modified Eagle Medium (DMEM), 2-[4-(2-Hydroxyethyl)-1-piperazinyl] ethanesulfonic Acid (HEPES) and Penicillin-Streptomycin Mixed Solution (Stabilized) were purchased from Nacalai Tesque (Japan). Fetal bovine serum (FBS) was purchased from JR Scientific Inc. (USA). Cell Titer 96® Nonradioactive Cell Proliferation Assay kit was purchased from Promega (USA). IL-6 (mouse) enzyme immunoassay kit was purchased from Cayman (USA).

Mammalian protein extraction reagent (M-PER) and PGE-2 competitive ELISA kit were purchased from Thermo Fisher Scientific Inc. (USA). Complete protease inhibitor cocktail tablets were purchased from Roche (Germany). Antibodies against COX-2 and beta actin were purchased from Abnova (Taiwan). DAB substrate was purchased from Rockland (USA).

### Extraction method

Pomegranate extract fractions were prepared as described by Ben Saad and Kim KH [19]. The whole fruits were washed with water and fruits with cuts were discarded, then the fruits were freeze-dried and ground into dry powder using a Waring blender. Then the powder was dried in an oven at 40 °C for 24 h. The powder was sieved through 24 mesh filter. The resultant powder of 1 kg was extracted with 2500 mL of 80% ethanol in water at room temperature for 24 h in a shaking water bath and filtered with whatman filter paper. The ethanol was removed by using RV10 rotary evaporator (IKA, Guangzhou, China) and the resultant residue was 110 g of crude ethanol pomegranate extract (EPE) yield 11%. Approximately, 100 g of crude EPE was mixed with 200 mL of n-hexane to dissolve the non-polar compounds. The mixture was filtered with whatman filter paper and the filtrate was concentrated by the rotary evaporator yielding the hexane fraction. The hexane insoluble residue was subjected to partitioning between the ethyl acetate and distilled water (immiscible solvents) using a separatory funnel. The ethyl acetate was removed using rotary evaporator and the water was removed by a freeze dryer to give the EtOAc fraction and the water fraction. The amount of EtOAc fraction was 42.2 g yield 3.5%.

### Phytochemical analysis

#### Isolation and structure identification of ellagic acid and punicalagin A&B from EtOAc by HPLC and MS analysis with spectrometry

The EtOAc sample was prepared and run on Shimadzu HPLC with UV detector. Standards were used to establish the retention time of interest. Pomegranate ethyl acetate extract (500 mg) was loaded in preparative HPLC. The standard and sample of interest were separated on a Luna Phenyl Hexyl, 250 × 4.6 mm, 5 um column was employed at ambient temperature using the following method mobile Phase A: 0.1 formic acid in water, mobile phase B: 100% methanol with the following gradient 0.01–3 min, A95%, B5%, 15–20 min A50% B50%, 25 min, A 10% B90%, 27–30 min A95% B5% with the flow rate 1.0 mL/min, injection volume 10.0 μL. The identification of ellagic acid and punicalagin A&B was made by comparing to standards. LC-MS Waters was used for mass detection. Gallic acid was isolated from EtOAc by another method previously reported by Ben Saad and Kim, 2015 [19].

#### Cell culture

Frozen RAW264.7 cells were cultured in dulbecco’s high Glucose modified Eagles medium (DMEM) supplemented with 10% of FBS, 4 mM l-glutamine, 110 mg/L sodium pyruvate, 25 mM HEPES, 100 U/mL of penicillin and 100 μg/mL of streptomycin. Cells were maintained in an incubator at 37 °C with 5% CO_2_ and 95% humidity until 80% confluent was achieved in 75 cm^2^ flasks. The culture medium was replaced every 3 days. Subcultures were prepared by scraping as per recommended by ATCC. For use in cell culture experiments, the isolated compounds, ellagic acid, gallic acid and punicalaginA&B from EtOAc were prepared in DMEM with 1% DMSO. To study the dose–response relationship four doses 50, 100, 150 and 200 μg/mL were used. These doses were selected based on literature [20] and tests in our laboratory. Cell culture treated with each isolated compound plus LPS and LPS alone were analyzed.

#### Cell viability assay

The cell viability was carried out using the CellTiter 96® Nonradioactive Cell Proliferation Assay kit according to the manufacturer’s instructions. In brief, cells were seeded at 5 × 10^4^ cells/well in 160 μL of complete culture medium into a 96-well, flat-bottom cell culture plate, and incubated overnight. Twenty microliter of each isolated compound were then being added into the wells to final concentrations of 0, 50, 100, 150 and 200 μg/mL in triplicate, 2 h before addition of 20 μL LPS to a final concentration of 1 μg/mL, and incubated for another 24 h (end point of treatment). Fifteen microliter of pre-warmed dye solution was added into each well and further incubated for 4 h. At the end of incubation, the wells were mixed with 100 μL of the solubilization solution/stop mix solution. One hour later the absorbance was read using a 96-well plate reader at wavelength of 570 nm against reference wavelength of 690 nm.

#### Measurement of nitrite production

RAW264.7 cells were cultured in 96-well plates at 5 × 10^4^ cells/well and were treated for 24 h with each isolated compound (50–200 μg/mL) plus LPS (1 μg/mL) or LPS alone. For measurement of nitrite in culture media, Griess reagent (modified) was used. In brief, at the end point of treatment, 100 μl of cell culture medium of each well was collected and mixed with equal volume of 1× Griess reagent and incubated for 15 min in the dark then the absorbance at 540 nm was measured in a 96-well plate reader. Fresh culture medium was used as the blank.

#### Measurements of IL-6 and PGE-2 production

RAW264.7 cells were cultured and treated with each isolated compound plus LPS or just LPS as mentioned above. Culture media were collected at 6 h and 24 h post-LPS stimulation for IL-6 and PGE-2 measurements, respectively. Both IL-6 and PGE-2 in culture media were separately quantitated with IL-6 (mouse) enzyme immunoassay kit and PGE-2 competitive ELISA kit according to the manufacturers’ instructions.

#### Measurement of COX-2 production

RAW264.7 cells were cultured in 24-well plates at 5 × 10^5^ cells per well and were treated for 24 h with each isolated compound as previously mentioned. Cell lysate was prepared with M-PER and added to complete protease inhibitor cocktail tablet. The total protein concentration of cell lysate was determined by Bradford method using a protein assay kit. COX-2 production was measured from cell lysate using western blot approaches with anti-beta actin antibody as loading control [21].

Cell lysates were electrophoresed by SDS-PAGE, transferred to nitrocellulose membrane and then probed with anti-COX-2 antibodies at a dilution of 1:2000 in TBST for 3 h at room temperature followed by overnight incubation in 4 °C. Subsequently, the membrane was washed thoroughly and incubated with a secondary antibodies conjugated with HRP. The membrane was again washed thoroughly with and developed in DAB substrate for detection of bands.

### Statistical analysis

Each experiment was repeated at least three times and the results were expressed as mean ± SD. Statistical significances were compared between each treated group and analyzed by the using Dunnett’s post test of one way ANOVA and Tukey Kramer test keeping LPS: 1 ug/mL + isolate: 0 ug/mL as control. All graphs were generated using Graph Pad Prism 5.0 and data *p* < 0.05, *p* < 0.01 and *p* < 0.001 were considered significant and marked with *, ** and *** respectively.

## Results

### Phytochemical screening

**Fig. 1:**
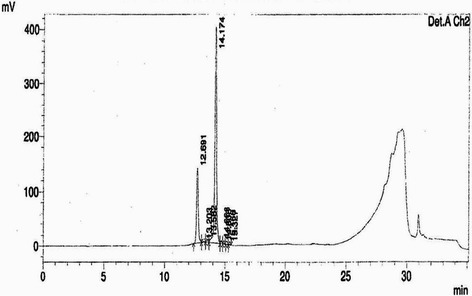
HPLC chromatogram of ethyl acetate fraction showing the presence of punicalagin A&B collected at 14.1 min

**Fig. 2:**
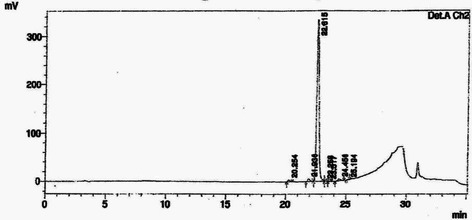
HPLC chromatogram of ethyl acetate fraction showing the presence of ellagic acid collected at 22.6 min

**Fig. 3:**
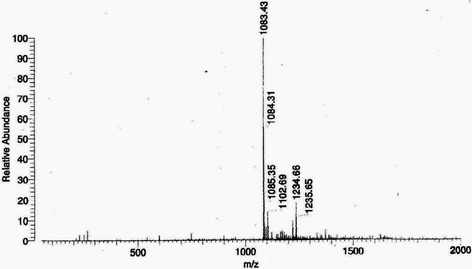
Mass spectrum showing the presence of punicalagin A&B [M-H] -*m/z* 1083.43

**Fig. 4:**
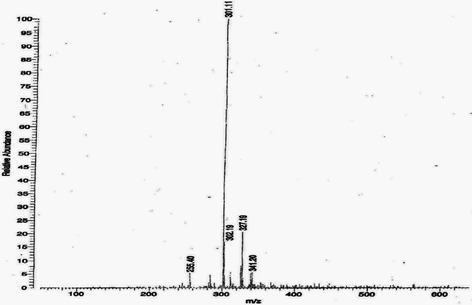
Mass spectrum showing the presence of ellagic acid [M-H] -*m/z* 301

The EtOAc fraction showed the presence of ellagic acid 67 mg/g collected at RT = 22.6 min, and punicalagin A&B 52 mg/g collected at RT = 14.1 min (Figs. 1 and 2). The punicalagin mass spectra was 1083 in negative mode and ellagic acid mass spectra was 301 in negative mode (Figs. 3 and 4). These results are consistent with a previous study in which pomegranate juice polyphenols were identified using different experimental conditions [22]. It has also been reported that the single charge *m/z* 1083 ion was detected for punicalagin A&B and a single of charge *m/z* 301 ion was detected for ellagic acid [19]. Gallic acid was collected at RT = 17 min and mass spectra was 169 in negative mode as previously reported by BenSaad and Kim, 2015 [19].

**Fig. 5:**
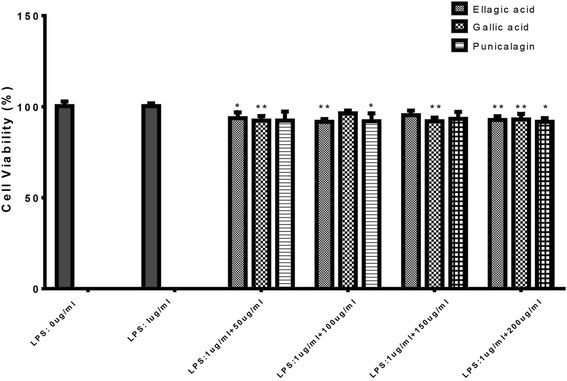
Cell viability was determined from the 24 h culture of cells stimulated with LPS (1 μg/mL) in the presence of ellagic acid, gallic acid and punicalagin The data represent mean ± SD of *n* = 3, *p < 0.05,**p < 0.01 vs LPS treated control

In this study, the isolated ellagic acid, gallic acid and punicaligin A&B were also examined for their effects on LPS-induced inflammation of the RAW264.7 cells. First, the cytotoxic effect of ellagic acid, gallic acid and punicaligin A&B on RAW264.7 cells were evaluated using the cell viability assay. MTT assay results for ellagic acid, gallic acid and punicaligin A&B showed a decrease in the cell viability (Fig. 5). The percentage of viable cells decreased, with a minimum of 87% survival, which still correlates to the non-toxic category. If 80% or higher survival rate shown in MTT reaction, it is determined that there is no cytotoxicity [23].

**Fig. 6:**
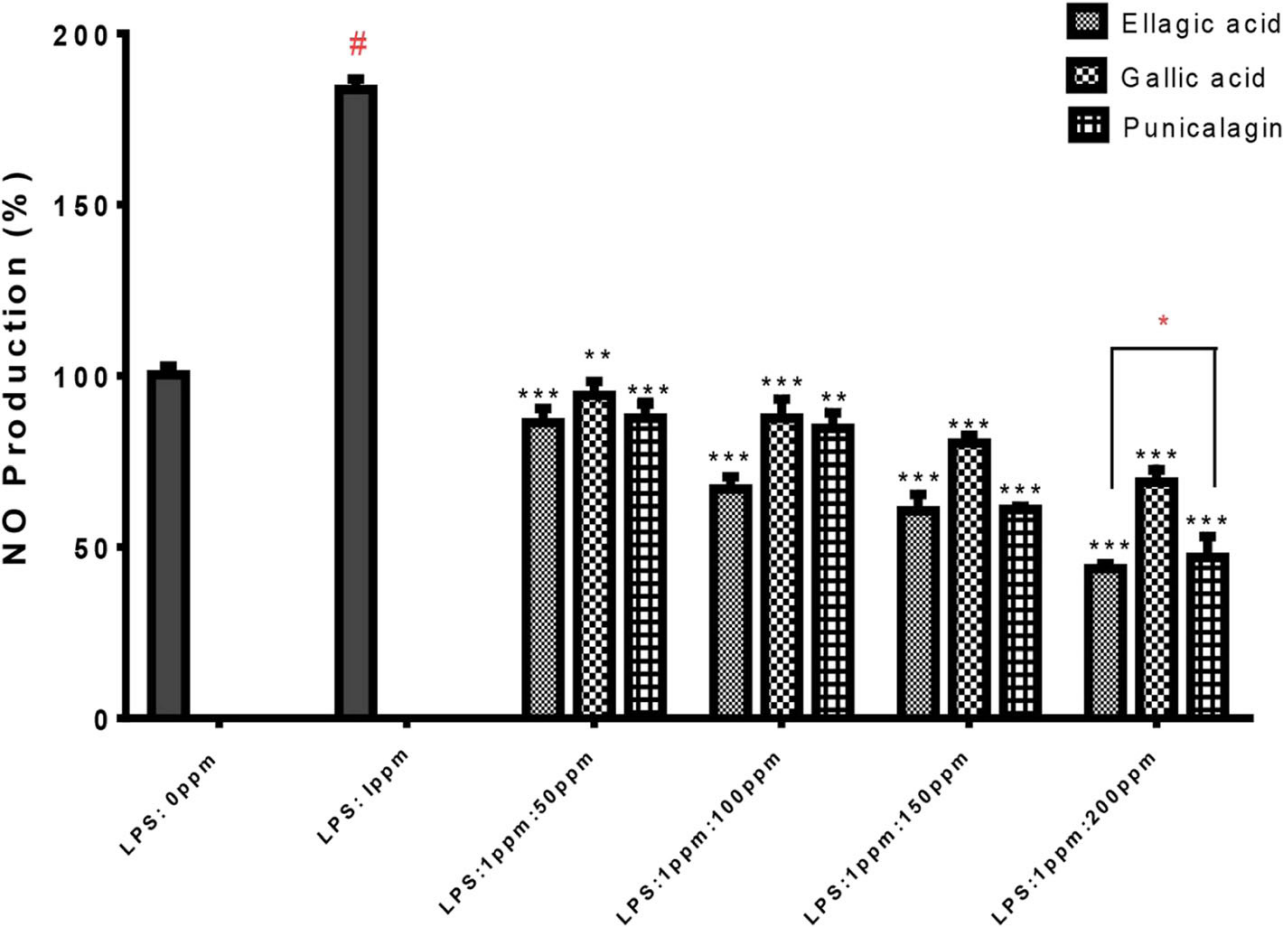
Effect of ellagic acid, gallic acid and punicalagin on nitrite production in LPS-stimulated RAW264.7 cells. The cells were stimulated with 1 μg/mL of LPS only or with LPS plus various concentrations (50, 100, 150, 200 μg/mL) of gallic acid for 24 h. Nitrite production was determined by the Griess reagent method. The data represent the mean ± SD of *n* = 3, *** *p* < 0.001, vs #LPS treated control* means significant difference by Tukey Kramer test

To investigate the effect of ellagic acid, gallic acid and punicalagin A&B on NO production, we measured the accumulation of nitrite, a stable oxidized product of NO in culture media by using Griess reagent method [23]. Nitrite production was examined in RAW264.7 cells stimulated with LPS in the presence or absence of ellagic acid, gallic acid and punicalagin A&B (50, 100, 150, 200 μg/mL) for 24 h. The nitrite production was inhibited by ellagic acid, gallic acid and punicalagin A&B treatments in a concentration–dependent manner (Fig. 6, and it was observed that three isolated compounds caused maximum inhibition of nitrite production at a concentration of 200 μg/ml) However, the highest inhibition effect was detected with ellagic acid in comparison to other two compounds.

**Fig. 7:**
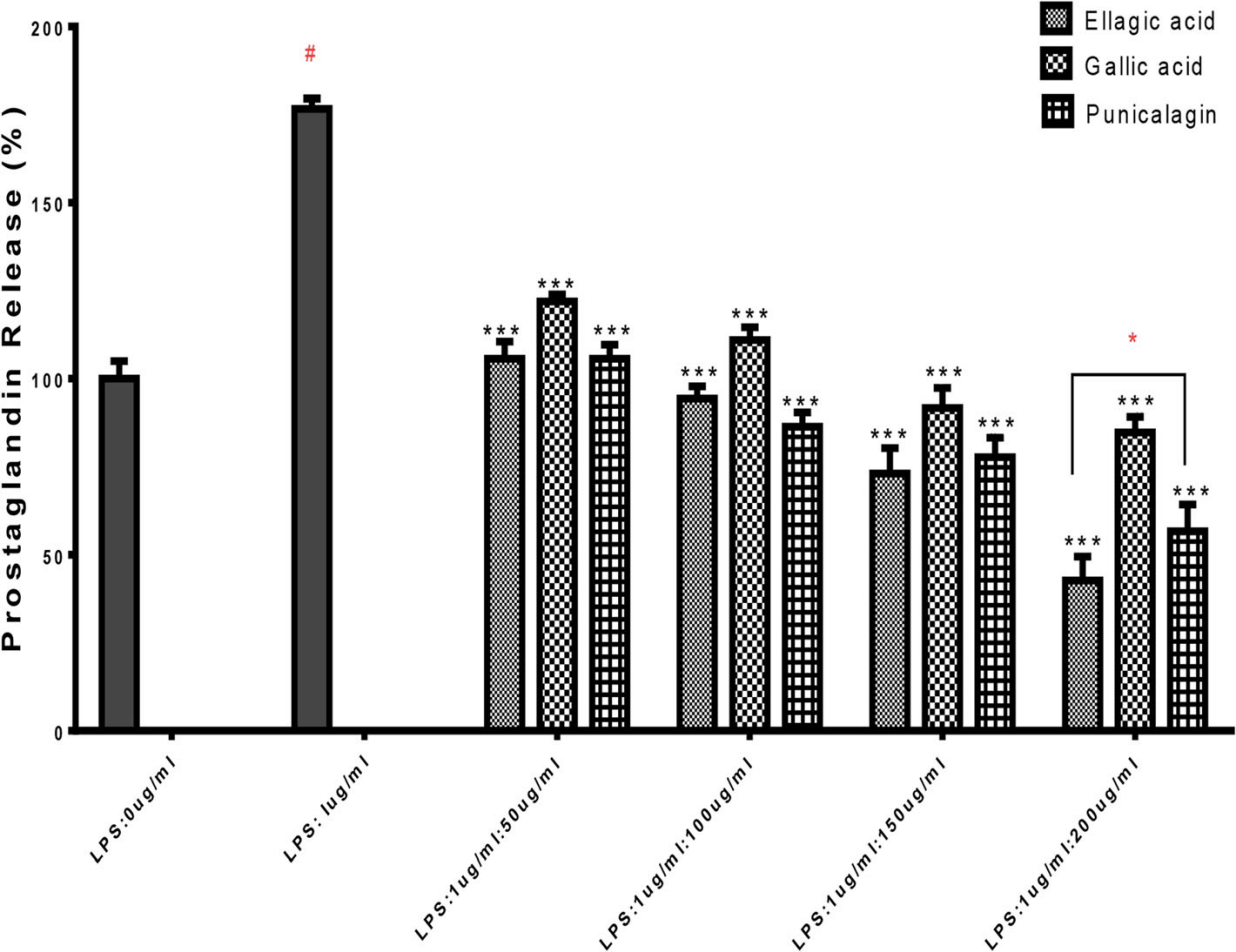
Effect of ellagic acid, gallic acid and punicalagin on PGE-2 production in LPS-stimulated RAW264.7 cells. The cells were stimulated with 1 μg/ml of LPS only or with LPS plus various concentrations (50, 100, 150, 200 μg/mL) of ellagic acid, gallic acid and punicalagin *n* = 3, ***p < 0.001 vs #LPS treated control* means significant difference by Tukey Kramer test

**Fig. 8:**
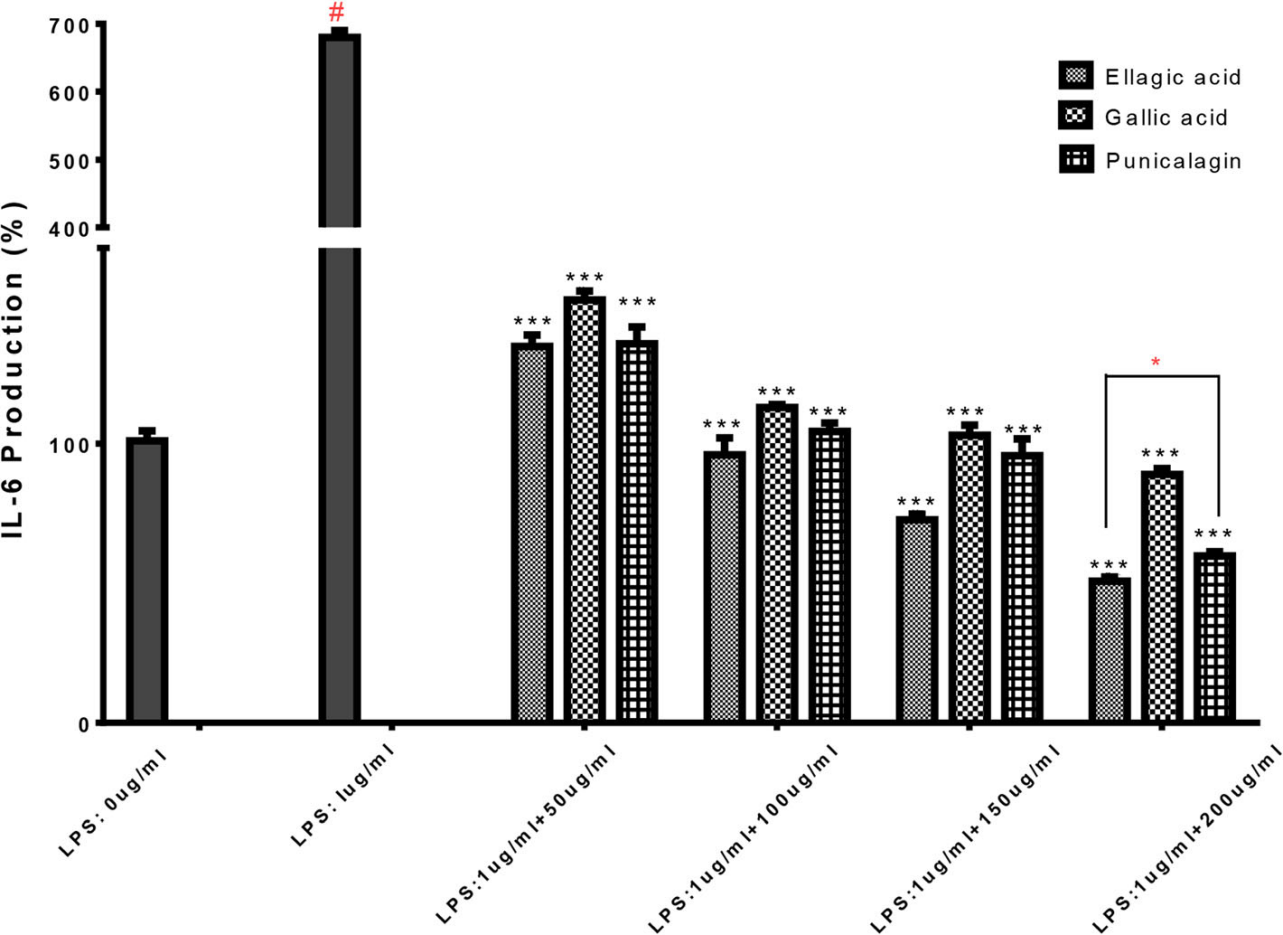
Effect of ellagic acid, gallic acid and punicalagin on IL-6 production in LPS-stimulated RAW264.7 cells. The cells were stimulated with 1 μg/ml of LPS only or with LPS plus various concentrations (50, 100, 150, 200 μg/mL) of for ellagic acid, gallic acid and punicalagin 6 h. IL-6 produced and released into the culture medium was assayed by ELISA method. The data represent the mean ± SD of *n* = 3, *** *p* < 0.001 vs #LPS treated control* means significant difference by Tukey Kramer test

The immune response to some inflammatory stimuli is known with the production of PGE-2 and interleukins therefore, we examined the effects of ellagic acid, gallic acid and punicalagin A&B on PGE-2 and IL-6 productions. The expressions of PGE-2 and IL-6 were measured in the medium of RAW264.7 cells cultured with LPS (1 μg/mL) in the presence or absence of isolated compounds (50, 100, 150, 200 μg/ml) for 24 h in case of PGE-2 and 6 h in case of IL-6. PGE-2 and IL-6 produced and released into the culture medium was assayed by ELISA method. As shown in (Figs. 7 and 8) ellagic acid, gallic acid and punicalagin A&B suppressed LPS-induced PGE-2 and IL-6 production in a dose concentration-dependent manner.

**Fig. 9:**
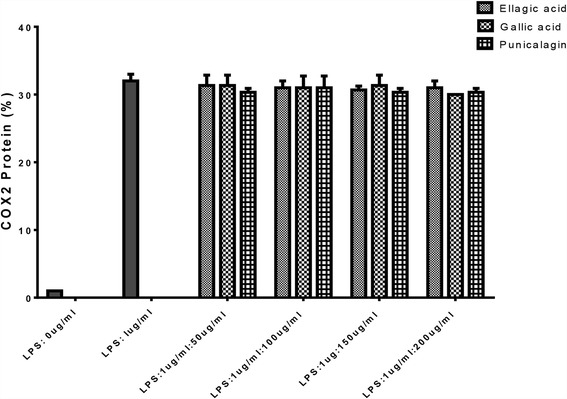
Effect of ellagic acid, gallic acid and punicalagin on the activation of COX-2 in the LPS-stimulated RAW264.7 cells. RAW264.7 cells were stimulated with LPS (1 μg/mL) in ellagic acid, gallic acid and punicalagin A&B (50, 100, 150, 200 μg/mL) for 24 h. Whole-cell lysates were subjected to 10% SDS-PAGE and expression of COX-2 and β-actin were determined by western blotting. Band intensities were expressed as relative density compared to LPS control. The figure is representative of three similar experiments

We further evaluated the effect of ellagic acid, gallic acid and punicalagin A&B on LPS-induced COX-2 gene expression in macrophages. The expression of COX-2 protein was measured in RAW264.7 cells exposed to LPS (1 μg/ml) for 24 h. Ellagic acid, gallic acid and punicalagin A&B did not suppress LPS-induced COX-2 expression. Western blotting band intensities were expressed as relative density compared to the untreated controls and measured by Image J software (National Institute of Health, US) (Fig. 9).

## Discussion

Feldmann and Kim research teams reported in 2008 that inflammation is a complex process regulated by a cascade of cytokines, growth factors, NO and prostaglandins produced by activated macrophages [24–26]. During inflammation, macrophages play a central role in the management of many different immunopathological phenomena, including the overproduction of pro-inflammatory cytokines and inflammatory mediators such as IL-1B, IL-6, NO, iNOS, COX-2 and TNF-alpha [24–26]. Our results showed that, ellagic acid, gallic acid and punicalagin A&B suppressed the levels of NO, PGE-2, IL-6 production in LPS-induced RAW264.7 cells.

Nitric oxide is a proinflammatory mediator that induces inflammation due to excessive production in pathological or abnormal physiological situations [27]. The synthesis of macrophage NO synthase enzyme, which generates NO from arginine is induced by LPS [28]. The increase in production of NO is harmful to the affected tissue, and may lead to acute or chronic inflammation [29]. Therefore, NO production inhibition in inflammation has a potential therapeutic significance. A previous study has reported that punicalagin isolated from a Chinese variety of *P.granatum* suppressed NO production in RAW267.4 cells stimulated macrophages [20]. Our study did confirm that punicalagin A&B effectively decreased NO production using the same cell line. In addition, we have shown in this study that ellagic acid and gallic acid reduced NO production in RAW267.4 cells treated with LPS.

The isolated compounds ellagic acid, gallic acid and punicalagin A&B inhibited the PGE-2 production dose dependently.

COX-2 is an enzyme that generates prostaglandins, which is induced by proinflammatory cytokines and other activators, such as LPS, resulting in the release of a large amount of PGE2 at inflammation sites. Therefore, identification of COX-2 inhibitors is considered to be a promising approach to protecting against inflammation. In this study we found that isolated compounds ellagic acid, gallic acid and punicalagin A&B inhibited the PGE-2 production dose dependently. A recent study on punicalagin [30] produced similar findings.

Another recent study reported that punicalagin mediated inhibition of PGE2 production in LPS-stimulated macrophage cells was associated with down regulation of COX-2 proteins [31].

However, in our study COX-2 protein expression of LPS-induced RAW264.7 cells was not affected after treatment with the ellagic acid, gallic acid and punicalagin A&B for 24 h. Similar findings has been reported when four isolated compounds namely, punicalagin, punicalin, strictinin A and granatin B which were hydrolysable tannins from *P. granatum* did not reduce COX-2 protein expression after treatment for 18 h [20]. However, in the same study punicalagin was reported to inhibit COX-2 expression after treatment period of 8 h. Their findings have indicated that punicalagin may inhibit the enzyme at an early stage but not a late stage.

IL-6 has been proven to be a key player in chronic inflammation, and IL-6 levels are elevated in inflammatory diseases in humans [32]. In this research project, the three isolated compounds gallic acid, ellagic acid and punicalagin effectively decreased IL-6 dose dependently. Importantly, this agrees with a recent study where investigators isolated punicalagin and showed that punicalagin did reduce IL-6 levels in LPS induced macrophages [30]. The present study is the first showcasing the anti-inflammatory effects of isolated ellagic acid and gallic acid from *P. granatum*. However, scientific, evidence shows that ellagic acid and gallic acid from different sources other than *P. granatum* did inhibit NO, PGE-2 and IL-6 in different systems [29, 33, 34].

The results of the present study showed the anti-inflammatory potential of *P. granatum*. EtOAc was able to significantly, inhibit the inflammatory mediators NO, PGE2, and IL-6. The three isolated compounds ellagic acid, gallic acid and punicalagin A&B inhibited NO, PGE2, and IL-6 in LPS – induced RAW 267.4 macrophages. Therefore, we postulate that ellagic acid, gallic acid and punicalagin A&B may be the compounds responsible for *P.granatum* anti-inflammatory effect. Recent studies have reported the antiinflammatory effect of punicalagin [20, 30, 31] yet we are first to report the antiinflammatory effect of gallic acid and ellagic acid, whether these compounds work as sole agents or have a synergistic effect still remains a question.

Further studies, are required to investigate the combination of ellagic acid gallic acid and punicalagin activity and also to investigate whether other compounds are involved in its anti-inflammatory effect. According to our results, we suggest that *P. granatum* (pomegranate) is a nutraceutical with a well-defined anti-inflammatory potential.

## Conclusion

The results obtained in this study clearly indicate that ellagic acid, gallic acid and punicalagin A&B isolated from *P. granatum* inhibited the production of NO, PGE2, and IL-6 in LPS–induced RAW267.4 macrophages. Therefore, we postulate that ellagic acid, gallic acid and punicalagin A&B may be the compounds responsible for the anti-inflammatory effect of *P. granatum*. However, there are some limitations in this study; positive controls have not been used. In addition, there are still differences between in vitro and in vivo system during agent testing which make it not totally reliable. Therefore, further studies are required to indicate if the results obtained in our study are relevant to human health.

## Abbreviations

COX-2: Cyclooxygenase-2
EtOAc: Ethyl acetate fraction of *P.granatum*
IL-6: Interleukin-6
LPS: Lipopolysaccharide
NO: Nitrous oxide
PGE: Prostaglandin- E
